# Decreased default mode network functional connectivity with visual processing regions as potential biomarkers for delayed neurocognitive recovery: A resting-state fMRI study and machine-learning analysis

**DOI:** 10.3389/fnagi.2022.1109485

**Published:** 2023-01-06

**Authors:** Zhaoshun Jiang, Yuxi Cai, Songbin Liu, Pei Ye, Yifeng Yang, Guangwu Lin, Shihong Li, Yan Xu, Yangjing Zheng, Zhijun Bao, Shengdong Nie, Weidong Gu

**Affiliations:** ^1^Department of Anesthesiology, Huadong Hospital Affiliated to Fudan University, Shanghai, China; ^2^Shanghai Key Laboratory of Clinical Geriatric Medicine, Huadong Hospital Affiliated to Fudan University, Shanghai, China; ^3^School of Health Science and Engineering, University of Shanghai for Science and Technology, Shanghai, China; ^4^Department of Radiology, Huadong Hospital Affiliated to Fudan University, Shanghai, China; ^5^Department of Geriatric Medicine, Huadong Hospital Affiliated to Fudan University, Shanghai, China; ^6^Research Center on Aging and Medicine, Fudan University, Shanghai, China

**Keywords:** whole-brain functional connectivity, default mode network, delayed neurocognitive recovery, machine learning, visual processing

## Abstract

**Objectives:**

The abnormal functional connectivity (FC) pattern of default mode network (DMN) may be key markers for early identification of various cognitive disorders. However, the whole-brain FC changes of DMN in delayed neurocognitive recovery (DNR) are still unclear. Our study was aimed at exploring the whole-brain FC patterns of all regions in DMN and the potential features as biomarkers for the prediction of DNR using machine-learning algorithms.

**Methods:**

Resting-state functional magnetic resonance imaging (fMRI) was conducted before surgery on 74 patients undergoing non-cardiac surgery. Seed-based whole-brain FC with 18 core regions located in the DMN was performed, and FC features that were statistically different between the DNR and non-DNR patients after false discovery correction were extracted. Afterward, based on the extracted FC features, machine-learning algorithms such as support vector machine, logistic regression, decision tree, and random forest were established to recognize DNR. The machine learning experiment procedure mainly included three following steps: feature standardization, parameter adjustment, and performance comparison. Finally, independent testing was conducted to validate the established prediction model. The algorithm performance was evaluated by a permutation test.

**Results:**

We found significantly decreased DMN connectivity with the brain regions involved in visual processing in DNR patients than in non-DNR patients. The best result was obtained from the random forest algorithm based on the 20 decision trees (estimators). The random forest model achieved the accuracy, sensitivity, and specificity of 84.0, 63.1, and 89.5%, respectively. The area under the receiver operating characteristic curve of the classifier reached 86.4%. The feature that contributed the most to the random forest model was the FC between the left retrosplenial cortex/posterior cingulate cortex and left precuneus.

**Conclusion:**

The decreased FC of DMN with regions involved in visual processing might be effective markers for the prediction of DNR and could provide new insights into the neural mechanisms of DNR.

**Clinical Trial Registration:**

: Chinese Clinical Trial Registry, ChiCTR-DCD-15006096.

## 1. Introduction

Delayed neurocognitive recovery (DNR) is a common complication of the neurological system, whose incidence is about 25.8% after non-cardiac surgery in elderly patients (Moller et al., 1998; Evered et al., 2018). With the development of an aging society, more and more geriatric surgical patients suffer from adverse outcomes caused by cognitive dysfunction after surgery, such as prolonged recovery time and increased incidence of complications (Evered et al., 2017; Chen and Qin, 2021). Hence, it is of crucial importance to understand the underlying pathological mechanisms of DNR and identify the potential biomarkers of DNR for its prevention.

In most brain function studies, resting-state functional magnetic resonance imaging (rs-fMRI) served as a useful approach to describe the connection characteristics of the brain networks associated with certain disease states (Rubinov and Sporns, 2010; Wu et al., 2021). Of particular interest is the default mode network (DMN), which is the most representative, stable, and individual-identifiable network in the resting state, as well as the most studied one in Alzheimer’s disease (AD) and mild cognitive impairment (MCI; Li et al., 2022). The DMN is not only engaged in the integration of cognitive, but also in memory recollection and reconstruction of long-term memories during retrieval (Krajcovicova et al., 2017). The medial prefrontal cortex (mPFC) and posterior cingulate cortex (PCC) are the core hubs of DMN. Besides, the lateral parietal cortex, middle cingulate cortex (MCC), and hippocampal gyrus are also components of DMN. These specific regions exhibit highly organized resting functional activity. However, most of the existing studies exclusively focused on the FC of mPFC and PCC, thus overlooking a lot of information and details about the DMN whole connectivity patterns. Hence, in the present study, we proposed a feasible approach to evaluate changes in whole-brain connectivity across all regions of DMN in patients with and without DNR by defining the ROI mask from the DMN atlas.

Machine learning can incorporate diversified rs-fMRI variables and identify potential biomarkers to build corresponding classification models (Solomon et al., 2020). As the information obtained from rs-fMRI data may provide objective evidence for the classification of DNR, combining it with machine learning algorithms may help improve the accuracy of early prediction of DNR. Rs-fMRI data mining has various stages, including rs-fMRI data preprocessing and analysis, extracted rs-fMRI features standardization (data preparation), model selection (machine-learning algorithms), the training and evaluation of models, parameter adjustment, and finally prediction (Sayadi et al., 2022).

In the present study, we used all subregions of DMN as the ROIs to determine whether DNR patients show abnormal FC between DMN and other voxels of the whole brain before surgery. Second, we adopted the support vector machine (SVM), logistic regression, decision tree, and random forest algorithms to develop machine-learning classification models to identify DNR patients based on rs-fMRI data. We hypothesized that whole-brain FC changes of DMN could serve as effective biomarkers to predict patients with DNR, which might help us to better understand the neural mechanisms of DNR and provide support for future clinical early screening and intervention in DNR patients.

## 2. Materials and methods

### 2.1. Ethics approval and participants

This nested case–control study was approved by the Ethics Committee of Huadong Hospital Affiliated to Fudan University (approval number: 20170020). The trial was registered on the Chinese Clinical Trial Registry^1^ with the identification number of ChiCTR-DCD-15006096. The principal investigator was Weidong Gu, and the date of registration was 16 March 2015.

Subjects were recruited at the Huadong Hospital Affiliated to Fudan University between September 2017 and February 2019. Written informed consent was obtained from all subjects participating in the trial before enrollment. The inclusion and exclusion criteria of our study population have been previously described (Jiang et al., 2020, 2021). The inclusion criteria were as follows: (1) patients scheduled to undergo non-cardiac surgery; (2) age ≥60 years old; (3) American Society of Anesthesiologists (ASA) classification I-III; and (4) right-handedness. The exclusion criteria were as follows: (1) education duration <6 years; (2) baseline mini-mental state examination (MMSE) score <24 points; (3) preexisting mental or psychiatric disease, cardiac or central nervous system vascular disease, Parkinson’s disease, cardiac or cranial surgery history; (4) major surgery within the past 12 months; (5) taking sedatives or antidepressants during the nearest year; (6) alcohol or drug abuse; and (7) vision and audition impairment or speech issues impeding communication.

### 2.2. Neurocognitive assessment

The Z-score method recommended by the International study of postoperative cognitive dysfunction (IPSOCD1) was used to identify the DNR (Moller et al., 1998). The comprehensive neurocognitive tests were conducted before surgery and at 7–14 days after the surgery for each subject. The neurocognitive tests consisted of MMSE, digit symbol substitution test (DSST), digit span forward and backward test (DSF/DSB), trail-making test-part A (TMT-A), and verbal fluency test (VFT). The Z score for each test and the composite Z score were calculated (Jiang et al., 2021). A patient was diagnosed with DNR when the Z scores of at least two of the neurocognitive tests or the composite Z score were ≥1.96.

### 2.3. Rs-fMRI data acquisition and preprocessing

Siemens Skyra 3.0 T MRI scanner was used to obtain all MRI data before surgery. The complete MRI acquisition protocol included three-dimensional (3D) anatomical T1-weighted imaging and fMRI echo-planar imaging. The 3D anatomical T1-weighted imaging parameters were as follows: 176 sagittal slices, repetition time = 1,900 ms, echo time = 3.57 ms, voxel size = 1 × 1 × 1 mm, and flip angle = 9°. The echo-planar imaging sequence parameters were as follows: 33 axial slices, slices thickness = 4 mm with a 0-mm gap, repetition time = 3,000 ms, echo time = 30 ms, voxel size = 3.4 × 3.4 × 4 mm, and flip angle = 90°. During the fMRI imaging, 120 volumes were obtained that lasted 8.5 min.

All rs-fMRI data were processed using Statistical Parametric Mapping version 12 and RESTplus version 1.24, based on MATLAB version R2013b. The preprocessing of rs-fMRI data consisted of eight following steps, where the first step included discarding the first five volumes to avoid the potential noise related to the participants’ adaptation to the scanner. Then, the remaining volumes were subjected to the slice-timing correction, head motion correction, spatial normalization, smoothing with a 6 × 6 × 6 mm Gaussian kernel, low-frequency filtering (0.01–0.08 Hz), linear trend of time course removal, and nuisance covariates regression (motion artifact, white matter signal, and cerebrospinal fluid signal; Jiang et al., 2021).

### 2.4. Functional connectivity analysis of DMN

A seed-based FC analysis was performed to explore the preoperative whole-brain voxel-wise FC alteration of the DMN in DNR patients. Nine regions in the dorsal DMN and nine regions in the ventral DMN were used as ROIs based on the DMN atlas from the Functional Imaging in Neuropsychiatric Disorders Lab at Stanford University (Shirer et al., 2012). Nine ROIs in the dorsal DMN included mPFC/anterior cingulate cortex, right superior frontal gyrus (SFG), PCC, MCC, left and right angular gyrus, left and right hippocampus, and thalamus. Nine ROIs in the ventral DMN included left and right retrosplenial cortex (RSC)/PCC, left and right middle frontal gyrus, left and right parahippocampal gyrus, left and right middle occipital gyrus, and precuneus.

The time series of each region were averaged and correlated with every other voxel within the gray-matter mask. The FC correlation maps were converted using Fisher’s r-to-z transform. A two-sample *t*-test was performed to explore the differences in seed-based FC between the DNR patients and non-DNR patients. The statistical maps were thresholded at *p* < 0.001 at the voxel level and *p* < 0.05 at the cluster level by false discovery rate (FDR) corrected for age, sex, and education duration (Chumbley and Friston, 2009).

### 2.5. Machine learning modeling

The prediction models of DNR were established using the SVM, logistic regression, decision tree, and random forest algorithms based on the FC features. Considering that the eigenvalues of FC features differ greatly, Scikit-learn’s “Preprocessing.StandardScaler” function was used to convert FC features into standard normally distributed data with zero mean and unit variance (Cho et al., 2022). The dataset was randomly divided into a training and a testing set at a ratio of 7:3. To reduce selection bias or overfitting, a 10-fold cross-validation method was employed for internal validation in the training set. In addition, because of the minority of DNR patients in the data set, the “balanced” class weight mode in the machine-learning algorithms was conducted to automatically adjust weights inversely proportional to class frequencies in the input data.

We further adopted the grid search method to identify the optimal parameters of the established prediction models. The main parameter to be adjusted in the LinearSVM algorithm was the penalty coefficient C (Wang L. et al., 2021). The logistic regression algorithm adopted the L2 penalty term, and adjusted the parameters including the inverse of regularization strength C and the maximum number of iterations (max_iter). The maximum depth of the tree (max_depth), the minimum number of samples required to split an internal node (min_samples_split), and the minimum number of samples required to be at a leaf node (min_samples leaf) were the most important parameters of decision tree algorithm. The random forest algorithm was an extension of the bagging method that fits a number of decision tree classifiers on various sub-samples of the dataset and uses averaging to improve the predictive accuracy and control overfitting. In addition to the parameters of the decision tree algorithm, the number of trees in the forest (n_estimators) was also a parameter that needed to be adjusted in the random forest classifier. The machine-learning algorithms can provide the corresponding weight of each variable, thereby identifying the variables significantly influencing the classification model.

The mean and standard deviation (SD) of the classification accuracy, sensitivity, specificity, and area under the receiver operating characteristic curve (AUC) of each model were computed in 100 runs of repeated re-divided dataset randomly. For further model assessment, a permutation test was performed to assess the statistical significance of the classification accuracy. The permutation test was repeated 1,000 times (Tonga and Huangb, 2022). During each time, the classifier reallocated labels of DNR patients and non-DNR patients to the training data randomly and repeated the whole classification process. The *p* value was acquired after the entire permutation was finished. Finally, Welch’s analysis of variance (ANOVA) and *Post-hoc* analysis (Games–Howell test with a Tukey correction) were conducted to compare the classification accuracies of different models. The statistical analyses of machine learning algorithms were performed in Python.

### 2.6. Statistical analysis

IBM SPSS 22.0 and GraphPad Prism were used to perform demographical statistical analyses. Continuous variables were assessed with a two-sample *t*-test or Mann–Whitney test and were presented as mean ± SD or median (interquartile range, IQR). Categorical variables were assessed with Chi-squared or Fisher exact test. For neurocognitive tests, analysis of covariance (ANCOVA) was used to compare follow-up scores between the two groups, with baseline scores as covariance. In addition, the paired-sample *t*-test analysis was used to compare the tests before and after surgery in the DNR patients and non-DNR patients. *p* < 0.05 indicated statistical significance.

## 3. Results

### 3.1. Subject characteristics

The flowchart of patient enrollment is provided in Supplementary Figure 1. In the present study, 74 patients completed the rs-fMRI scan and the neurocognitive tests. A total of 16 patients were diagnosed with DNR at 7–14 days postoperatively. Also, the duration of education was significantly shorter in the DNR patients [median (IQR), 6 (6, 9)] compared to the non-DNR patients [median (IQR), 9 (9, 12)] (*p* = 0.002). No significant differences were detected in the other baseline characteristics, including age, sex, height, weight, body mass index, surgical history, and comorbidities between the two groups. In addition, there were no significant differences in the surgical duration and nature of surgery between the groups (Table 1).

**Table 1:** Baseline characteristics.

	DNR (*n* = 16)	Non-DNR (*n* = 58)	*p-*Value
Age (years)	63.5 (62.0, 67.0)	64.0 (61.0, 68.3)	0.598
Sex (male/female)	12/4	29/29	0.075
Education (years)	6 (6, 9)	9 (9, 12)	0.002
Height (m)	1.68 ± 0.08	1.65 ± 0.08	0.185
Weight (kg)	59.0 (50.0, 70.5)	60.0 (54.8, 70.0)	0.324
BMI ≥ 24 (*n*, %)	3 (18.8)	19 (32.8)	0.437
Surgical history (*n*, %)	6 (37.5)	28 (48.3)	0.444
Hypertension (*n*, %)	7 (43.8)	24 (41.4)	0.865
Diabetes mellitus (*n*, %)	1 (6.3)	7 (12.1)	0.835
Surgical duration (min)	120 (114, 166)	95 (70, 135)	0.055
Minimally invasive/open surgery	12/4	54/4	0.107

BMI, body mass index; DNR, delayed neurocognitive recovery.

### 3.2. Neurocognitive assessments

We investigated group differences in follow-up scores of neurocognitive tests using ANCOVA, wherein we controlled for baseline scores. Our results showed that the DNR patients exhibited significantly lower MMSE, VFT, DSF/DSB, and DSST follow-up scores and higher TMT-A follow-up scores (seconds) compared to the non-DNR patients (all *p* < 0.05, Table 2). Paired-samples *t*-test analysis revealed that the performance of postoperative DSST and TMT-A assessment was the opposite in the two groups. For DNR patients, the DSST score after surgery was significantly worse than before, and the TMT-A time after surgery was significantly longer than before surgery. However, non-DNR patients showed improved performance in DSST and TMT-A post-surgery compared with pre-surgery. These results indicated that the DNR patients had poor performance after non-cardiac surgery in many domains of cognitive function, especially in those assessed by DSST and TMT-A, such as attention, visual–spatial ability, visual scanning, visual search speed, executive function, and visual motor coordination.

**Table 2:** Neurocognitive assessments.

	DNR	Non-DNR	*p-*Value
MMSE—*baseline*	26.69 ± 2.09	27.12 ± 1.87	0.579
MMSE*—follow-up*	25.50 ± 2.90	27.09 ± 2.26	0.032*
VFT—*baseline*	14.94 ± 4.19	15.76 ± 3.01	0.472
VFT—*follow-up*	12.75 ± 4.96^#^	16.29 ± 3.81	0.002*
DSF*—baseline*	7.31 ± 1.74	7.67 ± 1.25	0.534
DSF—*follow-up*	6.75 ± 1.92^#^	7.74 ± 1.04	0.008*
DSB—*baseline*	4.13 ± 1.50	3.79 ± 0.97	0.563
DSB*—follow-up*	3.25 ± 0.86^#^	3.91 ± 0.90	<0.001*
DSST—*baseline*	23.44 ± 7.84	29.10 ± 10.90	0.038
DSST—*follow-up*	20.69 ± 7.44^#^	31.60 ± 10.83^#^	<0.001*
TMT-A—*baseline (s)*	62.69 ± 39.65	49.91 ± 21.29	0.245
TMT-A*—follow-up (s)*	76.38 ± 27.70^#^	45.55 ± 13.32^#^	<0.001*

Variables are presented as mean ± SD. ANCOVA, analysis of covariance; DNR, delayed neurocognitive recovery; DSB, digit span backward; DSF, digit span forward; DSST, digit symbol substitution test; MMSE, mini-mental state examination; SD, standard deviation; TMT-A, trail-making test-part A; VFT, verbal fluency test.

* A statistically significant difference using the ANCOVA (baseline score as a covariate), *p* < 0.05.

# A statistically significant difference compared with baseline score using the paired-samples *t*-test, *p* < 0.05.

### 3.3. Altered seed-based FC patterns of DMN

**Figure 1:**
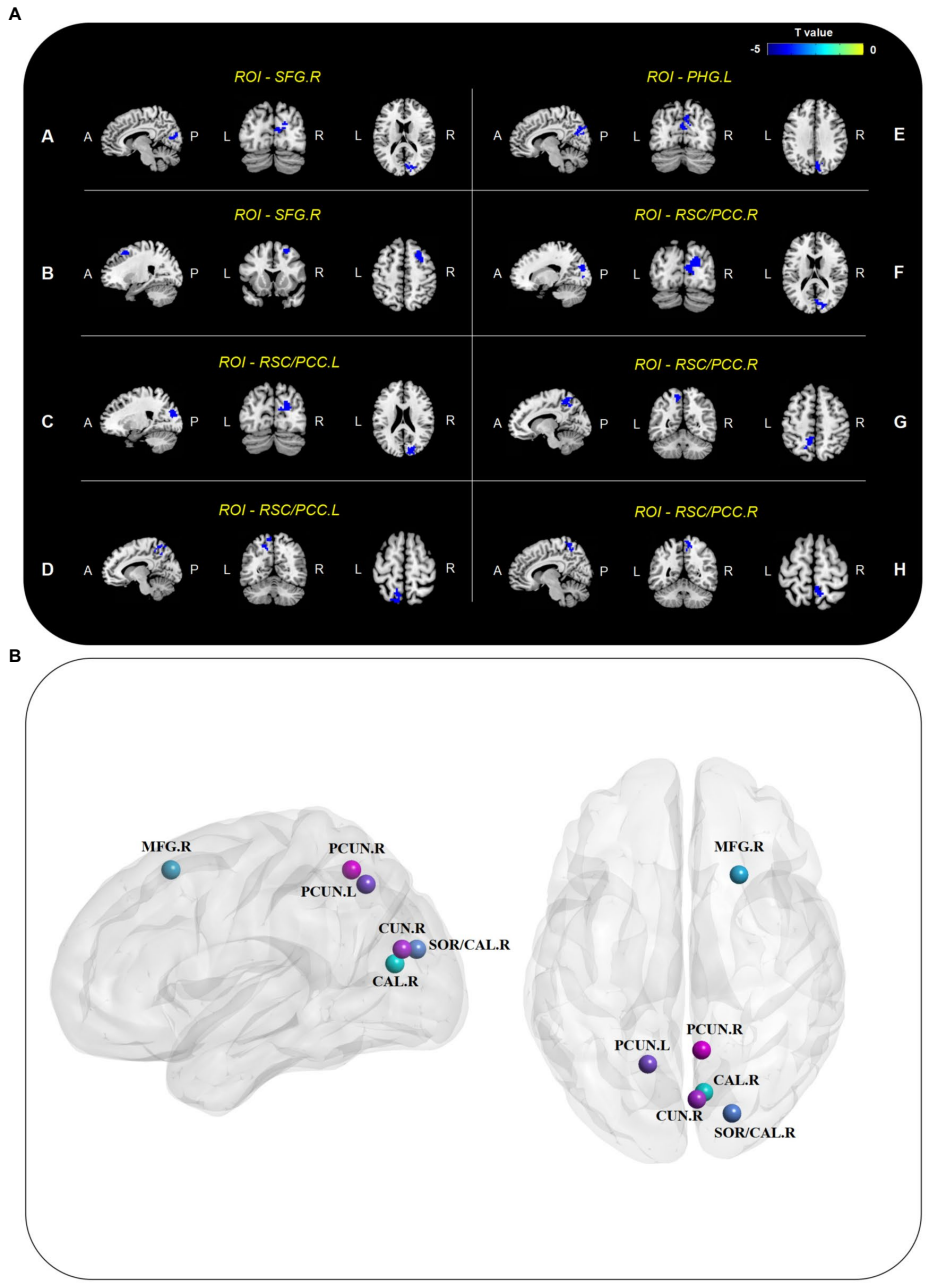
Altered seed-based whole-brain FC of DMN. **(A)** Brain regions with decreased connectivity to DMN core ROIs in the DNR patients compared to the non-DNR patients after adjusting for age, sex, and education duration (cluster *p* < 0.05, FDR-corrected). **(B)** Visualized nodes with peak T value of such brain regions. CAL, calcarine; CUN, cuneus; DMN, default mode network; DNR, delayed neurocognitive recovery; FC, functional connectivity; FDR, false discovery rate; MFG, middle frontal gyrus; PCC, posterior cingulate cortex; PCUN, precuneus; PHG, parahippocampal gyrus; ROIs, regions of interest; RSC, retrosplenial cortex; SFG, superior frontal gyrus; SOG, superior occipital gyrus; A, anterior; P, posterior; L, left; R, right.

Seed-based FC analysis revealed that the preoperative whole-brain resting-state connections of DMN core regions were significantly weakened in the DNR patients (Figure 1; Table 3). In the dorsal DMN, altered whole-brain FC was observed only in the right SFG. The DNR group exhibited decreased FC of the right SFG to the right calcarine and right middle frontal gyrus compared to the non-DNR group. Regarding the altered patterns of the ventral DMN, decreased connections were detected in the DNR group compared to the non-DNR group between the left parahippocampal gyrus and right cuneus. Also, the DNR group exhibited decreased FC of bilateral RSC/PCC to the right superior occipital gyrus/calcarine and left precuneus. In addition, decreased FC of the right RSC/PCC to the right precuneus was also found in the DNR patients. The results indicated that the key ROIs of DMN in DNR patients had altered resting-state functional connections with visual processing-related cortical areas, which mainly manifested as weakened connectivity. Age, sex, and education duration were included as covariates in the seed-based whole-brain FC analysis. The results were corrected using the cluster-based FDR (uncorrected voxel *p* < 0.001 and corrected cluster *p* < 0.05).

**Table 3:** Regions with decreased connectivity to DMN core ROIs in the DNR patients.

	ROIs	Brain regions	MNI coordinates (x/y/z mm)			Cluster size	Peak T
A	SFG.R	CAL.R	9	−72	15	88	−4.089
B	SFG.R	MFG.R	24	21	54	62	−4.816
C	RSC/PCC.L	SOG/CAL.R	21	−81	21	131	−5.054
D	RSC/PCC.L	PCUN.L	−15	−60	48	65	−4.264
E	PHG.L	CUN.R	0	−75	21	113	−4.111
F	RSC/PCC.R	SOG/CAL.R	21	−81	24	120	−4.397
G	RSC/PCC.R	PCUN.L	−15	−57	48	128	−4.519
H	RSC/PCC.R	PCUN.R	6	−54	54	58	−4.145

CAL, calcarine; CUN, cuneus; DMN, default mode network; DNR, delayed neurocognitive recovery; MFG, middle frontal gyrus; MNI, Montreal Neurological Institute; PCC, posterior cingulate cortex; PCUN, precuneus; PHG, parahippocampal gyrus; ROIs, regions of interest; RSC, retrosplenial cortex; SFG, superior frontal gyrus; SOG, superior occipital gyrus; R, right; L, left.

### 3.4. Machine learning prediction models

The resting-state FC data were standardized before training to ensure that machine learning estimators could correctly model all features. The mean and variance of each feature were converted to 0.00 and 1.00 by standardization. The data distribution before and after preprocessing is shown in Supplementary Figure 2. In order to develop the prediction model, the dataset was randomly divided into a training set containing 70% of the samples and a testing set containing the remaining 30%, whereas the models were validated using the 10-fold cross-validation method.

By the grid search method, the optimal penalty coefficient C of the SVM classifier was identified as 8.88889. The inverse of regularization strength C of 2 and max_iter of 10 were the optimal parameters of the logistic regression classifier. The optimal parameters of the decision tree classifier were max_depth of 9, min_samples_split of 2, and min_samples leaf of 2. And the optimal parameters of the random forest classifier were max_depth of 2, min_samples_split of 5, min_samples leaf of 2, and n_estimators of 20.

**Figure 2:**
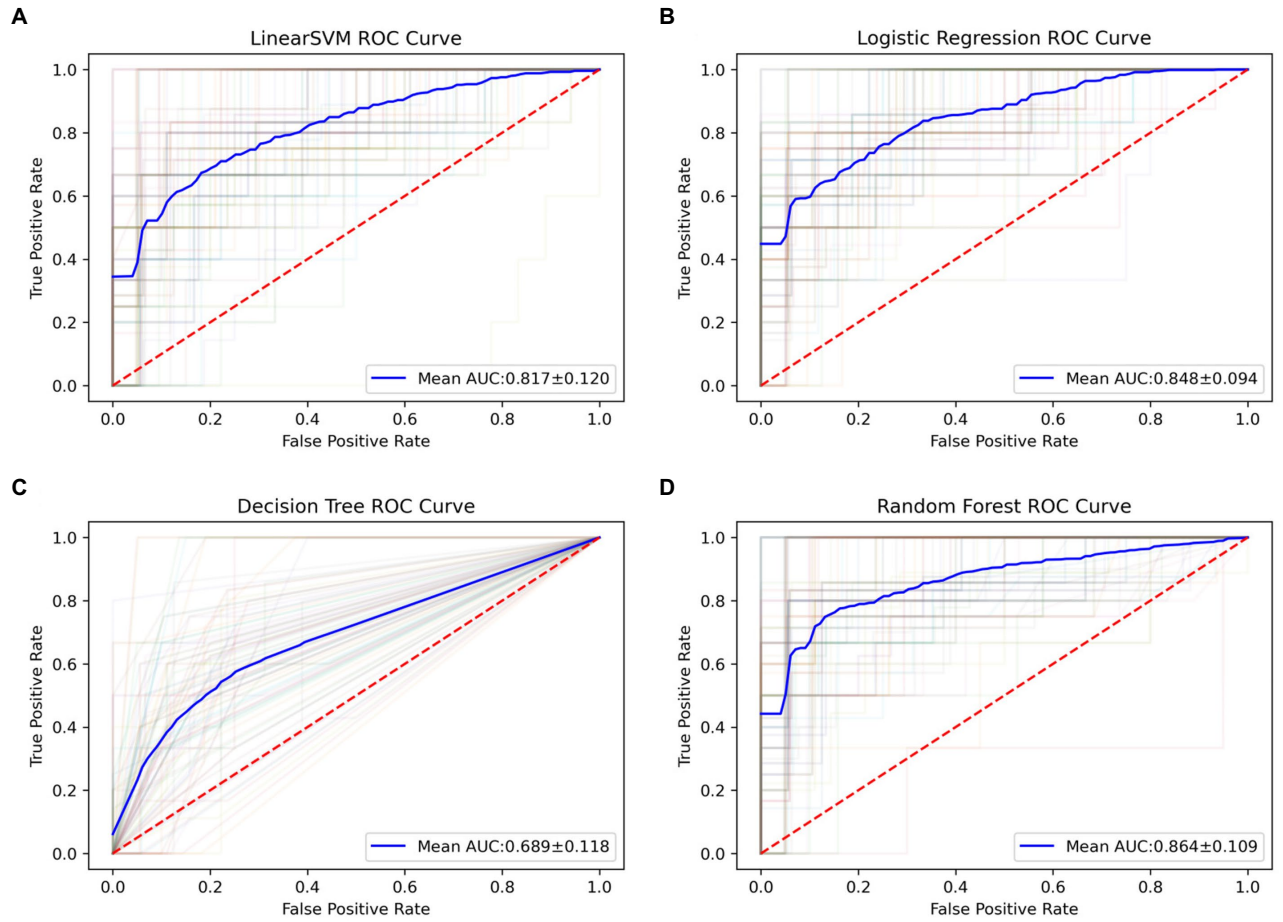
The ROC curves of all classification models in 100 runs of repeated re-divided dataset randomly. **(A)** The SVM model; **(B)** the logistic regression model; **(C)** the decision tree model; **(D)** the random forest model. AUC, an area under the curve; ROC, the receiver operating characteristic; SVM, support vector machine.

**Figure 3:**
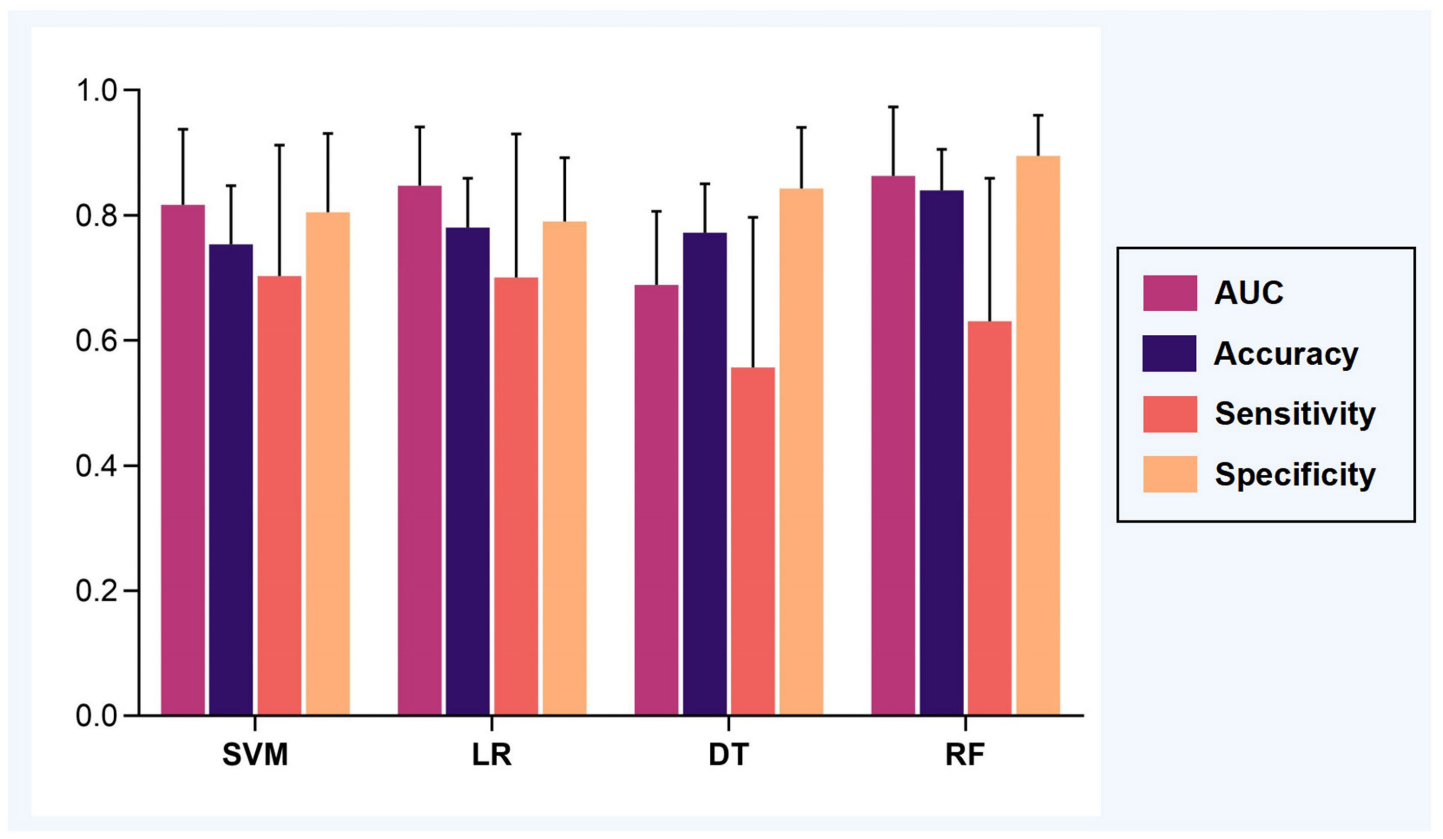
The classification performance of models. The mean AUC, accuracy, sensitivity, and specificity were calculated based on 100 runs of repeated re-divided dataset randomly. Abbreviations: AUC, an area under the curve; SVM, support vector machine; LR, logistic regression; DT, decision tree; RF, random forest.

Applying the 100 runs of repeated re-divided dataset randomly, the SVM classifier with optimal parameters achieved a classification accuracy of 75.3 ± 9.4%. The logistic regression classifier with optimal parameters achieved a classification accuracy of 78.1 ± 7.8%. The decision tree classifier with optimal parameters achieved a classification accuracy of 77.2 ± 7.8%. The random forest classifier with optimal parameters achieved a classification accuracy of 84.0 ± 6.5%. The AUC of all classification models is shown in Figure 2. The mean values and standard deviation of AUC, accuracy, sensitivity, and specificity of all classification models are summarized in Figure 3.

**Figure 4:**
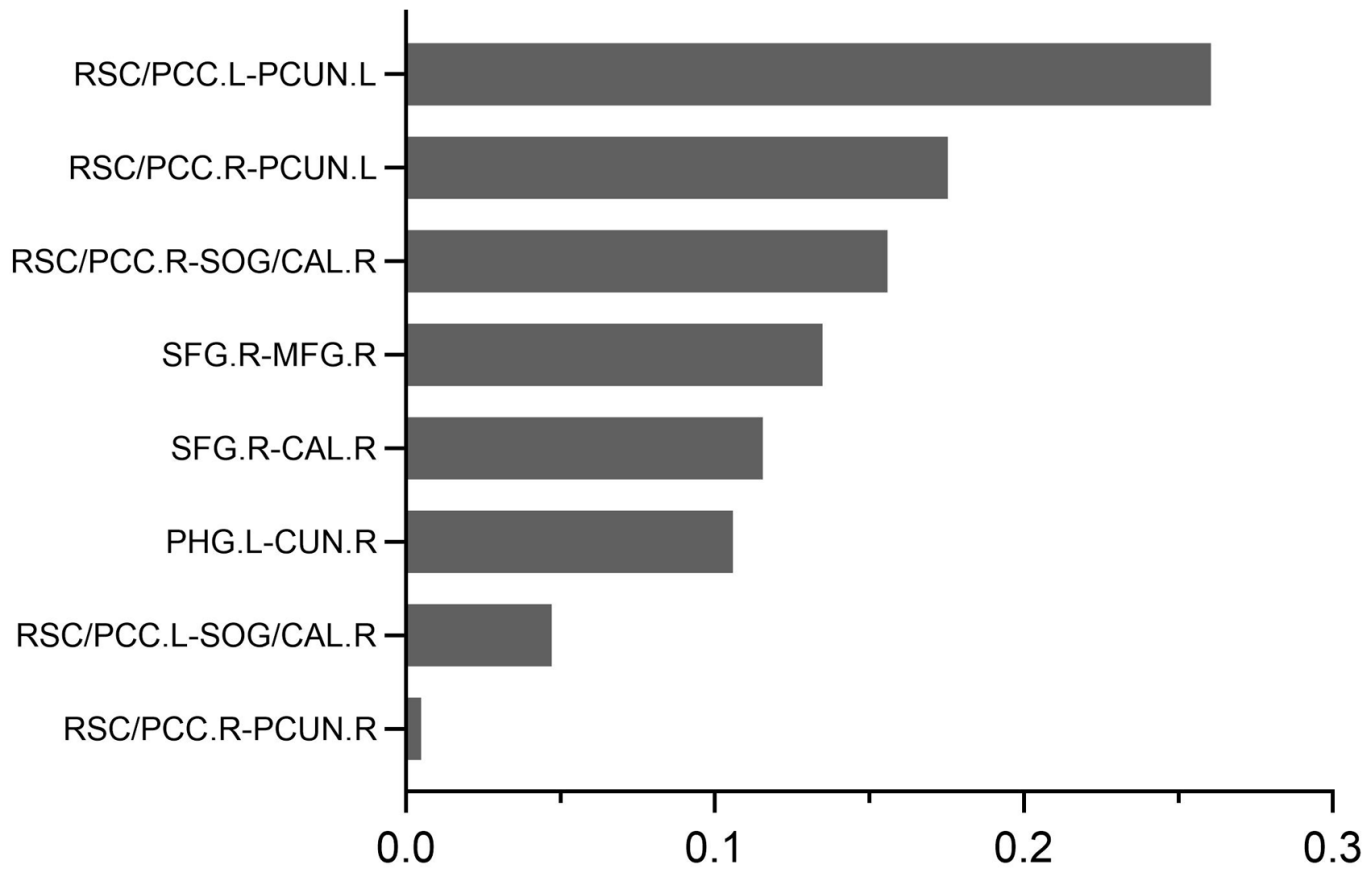
The feature weights of the random forest prediction models.

The permutation test can be seen that the SVM model (*p* = 0.022), the logistic regression model (*p* = 0.023), and the random forest model (*p* = 0.019) could distinguish the DNR group and the non-DNR group, whereas the decision tree model could not (*p* = 0.118). Furthermore, Welch’s ANOVA was performed to compare the classification accuracies of different models. It revealed significant differences among the SVM, logistic regression, and random forest models in the classification accuracies (Welch *F* = 34.002, *p* < 0.001). *Post-hoc* tests showed that the accuracy of the random forest model was higher than the SVM model (*p* < 0.001, 95% CI 0.060 to 0.114) and the logistic regression model (*p* < 0.001, 95% CI 0.035 to 0.084), while there was no significant difference in the accuracy between the SVM model and the logistic regression model (*p* = 0.068, 95% CI −0.002 to 0.056). The results indicated that the random forest classifier with optimal parameters could achieve the best prediction performance for DNR. The random forest algorithm also provided the corresponding weight of each feature, thereby identifying the features that influence the predictions (Corradi et al., 2018). Figure 4 shows the corresponding weights of each variable calculated by the random forest algorithm to reveal the contributions of these variables to the model.

## 4. Discussion

The results of the present study showed that the preoperative resting-state whole-brain FC of DMN features could be used to predict DNR by establishing a machine-learning model. The key findings are as follows: (1) the FC between the key ROIs of DMN and other brain regions, especially in visual processing-related cortical areas, in DNR patients before surgery was more weakened compared to non-DNR patients; (2) the random forest machine learning model showed that whole-brain FC features of DMN could be used to predict postoperative DNR with high accuracy.

The human brain can be viewed as an integrated network consisting of brain regions that are anatomically separated but functionally connected (van den Heuvel and Hulshoff Pol, 2010). Hence, compared with the activity of local brain regions, FC analysis can better reflect the actual operation mode of the whole brain. The FC approach has obtained good results in fMRI data classification studies (Wang et al., 2019; Dai et al., 2022). Recently, an increasing number of rs-fMRI studies have reported that resting-state FC before surgery has a primary role in predicting cognitive dysfunction after surgery. Our previous study found that patients with preoperative decreased FC between the bilateral MCC and left calcarine were more susceptible to DNR following non-cardiac surgery by logistic regression (Jiang et al., 2020). By exploring the whole-brain FC of key hubs in cognitive-related brain networks, we found that the altered whole-brain FC of DMN and central executive network could predict DNR in elderly patients using machine learning algorithms (Jiang et al., 2021). In addition, Wu et al. (2021) established a machine learning DNR classification model based on the multi-order brain FC network features, achieving good prediction performance. These rs-fMRI studies examined preoperative FC changes of brain networks in patients with cognitive decline after surgery, revealing some consistent findings, such as the importance of DMN connectivity.

The basal activity of the DMN is quite consistent during the resting state. The DMN has an essential role in episodic memory, processing of cognition and emotion, and so on (Shi et al., 2020). Functional abnormalities of DMN have been well-documented in various diseases related to cognitive impairment, such as AD and MCI (Zhang et al., 2020; Wang S. M. et al., 2021). Our preliminary findings suggested that the whole-brain FC of certain brain regions in the DMN was altered in DNR patients. Therefore, in the present study, we further explored the FC patterns of all components of DMN in DNR patients based on prior knowledge. We also found that the DMN connectivity was significantly decreased in DNR patients. Interestingly, the brain regions with altered DMN connectivity were predominantly in visual processing areas. Similar changes were detected in AD and MCI in previous studies. Krajcovicova et al. (2017) found that the patients with MCI/AD had disrupted PCC connectivity with areas of the ventral visual pathway, which suggested that the abnormal DMN connectivity patterns may provide a possible underlying mechanism of impaired visual processing in MCI/AD.

Consistent with cognitive disorders like AD and MCI, and in addition to memory deficits, impairment in other cognitive domains is present in DNR. Many studies used neuropsychological tests to demonstrate visual cognitive dysfunction in DNR patients (Lage et al., 2021; Ji and Li, 2022; Zhao et al., 2022). We also found that the DNR patients showed poor performance postoperatively in visual attention, visual memory, visual–spatial scanning, and visual search speed assessed by DSST and TMT-A. However, the current diagnosis methods of DNR, which are mainly based on assessments of neuropsychological tests, may easily be impacted by human factors. As the information obtained from rs-fMRI data could provide objective evidence for the brain activity characteristics of DNR, combining it with the manual assessment of DNR diagnosis may reduce the diagnosis errors of DNR and identify the potential biomarkers. Using different FC analysis methods, we demonstrated that the DNR patients showed abnormal connectivity in regions related to visual processing, especially decreased connection to the key regions in DMN. The altered MCC spontaneous activity in DNR patients was detected based on the Amplitude of Low-frequency Fluctuation (ALFF) analysis. In our previous study, MCC, a subregion of DMN, showed a significantly decreased FC to the calcarine in DNR patients by ALFF-based FC analysis (Jiang et al., 2020). In addition, we found that the other key region of DMN, the lateral parietal cortex, also exhibited a significantly decreased FC to the calcarine in DNR patients by seed-based FC analysis (Jiang et al., 2021). The calcarine lies in the center of the primary visual cortex and contributes to visual processing, such as visual attention and visual memory (Pratte and Tong, 2014; Bergmann et al., 2016). A large body of studies has suggested that abnormal calcarine FC might be associated with the occurrence and development of multiple cognitive disorders (Moon and Jeong, 2017; Zuo et al., 2017; Zhuang et al., 2019). Among the brain regions with abnormal FC to DMN identified in the present study, in addition to the calcarine, the cuneus, precuneus, and superior occipital gyrus, which are involved in visual processing, were identified. We hypothesized that the visual cognitive impairment in DNR patients was mainly caused by the inhibition of FC between the DMN and visual processing areas. The association of DMN and visual processing could be worthy of further study in future DNR research.

In recent years, machine learning has been widely used by cognitive dysfunction disease prediction studies due to its individualized classification ability (Mufti et al., 2019; Hu et al., 2021). The combination of brain network research based on fMRI and machine learning has become a topic of great interest. Yet, the data obtained by fMRI are highly dimensional, sample-limited, and non-linear. Traditional modeling methods or linear dimensionality reduction cannot meet the requirements of the model (Tan et al., 2022). Machine-learning algorithms such as SVM and random forest had advantages in dealing with small samples and non-linear features. In addition, since the fMRI original data contains a large amount of redundant information, selecting the proper feature subset is essential to avoid reducing the model’s performance (Shuai et al., 2017). The present study uses the two-sample *t*-test after FDR correction to extract features with intergroup differences. Based on the FC features of DMN selected by feature screening, we built a prediction DNR model using the random forest algorithm, achieving great performance.

There are some limitations in the present study. First, the postoperative FC patterns of DMN in DNR patients could not be identified because most subjects refused to perform another fMRI scan postoperatively. Second, the amount of experimental data is limited. A sufficient amount of data ensures the reliability and stability of machine learning. Although we have conducted a strict division of training and testing data and performed the SVM and random forest algorithm, which are suitable for small samples, the stability and reliability of the prediction models still need to be verified on more datasets, especially multi-center data.

In conclusion, the present study demonstrated that the decreased FC between the DMN and brain regions involved in visual processing was detected in elderly patients who developed DNR after non-cardiac surgery. Also, the characteristic FC patterns could be effective biomarkers for the prediction of DNR.

## Data availability statement

The raw data supporting the conclusions of this article will be made available by the authors, without undue reservation.

## Ethics statement

The studies involving human participants were reviewed and approved by Ethics Committee of Huadong Hospital Affiliated to Fudan University. The patients/participants provided their written informed consent to participate in this study.

## Author contributions

ZJ and YC made substantial contributions to the design, acquisition, analysis, and interpretation of study data, and drafted the manuscript. SLiu and PY made substantial contributions to the acquisition and analysis of study data, and provided critical revisions to the manuscript. YY made substantial contributions to the analysis and interpretation of study data, and provided critical revisions to the manuscript. GL made substantial contributions to the design and interpretation of study data, and provided critical revisions to the manuscript. SLi, YX, and YZ made substantial contributions to the acquisition of study data, and provided critical revisions to the manuscript. ZB made substantial contributions to the design of the study, and provided critical revisions to the manuscript. SN and WG made substantial contributions to the conception, design, acquisition, analysis, and interpretation of study data, drafted the manuscript, and provided critical revisions to the manuscript. All authors contributed to the article and approved the submitted version.

## Funding

This study was supported by the project of National Natural Science Foundation of China (82271286), Science and Technology Commission of Shanghai Municipality (20Y11900200), the Shanghai Municipal Health Commission (2020YJZX0119), Hospital research project of Huadong Hospital (2019jc016), the National Key R&D Program of China (2018YFC2002000), and the Shanghai Municipal Key Clinical Specialty (shslczdzk02801).

## Conflict of interest

The authors declare that the research was conducted in the absence of any commercial or financial relationships that could be construed as a potential conflict of interest.

## Publisher’s note

All claims expressed in this article are solely those of the authors and do not necessarily represent those of their affiliated organizations, or those of the publisher, the editors and the reviewers. Any product that may be evaluated in this article, or claim that may be made by its manufacturer, is not guaranteed or endorsed by the publisher.

## Footnotes

^1^ http://www.chictr.org.cn
