# Supplementary material for: Decreased default mode network functional connectivity with visual processing regions as potential biomarkers for delayed neurocognitive recovery: A resting-state fMRI study and machine-learning analysis
Source: Front Aging Neurosci. 2023 Jan 6;14:1109485. doi: 10.3389/fnagi.2022.1109485 (PMC9853194; doi:10.3389/fnagi.2022.1109485)
Supplement: Supplementary file 1 [file Data_Sheet_1.docx]

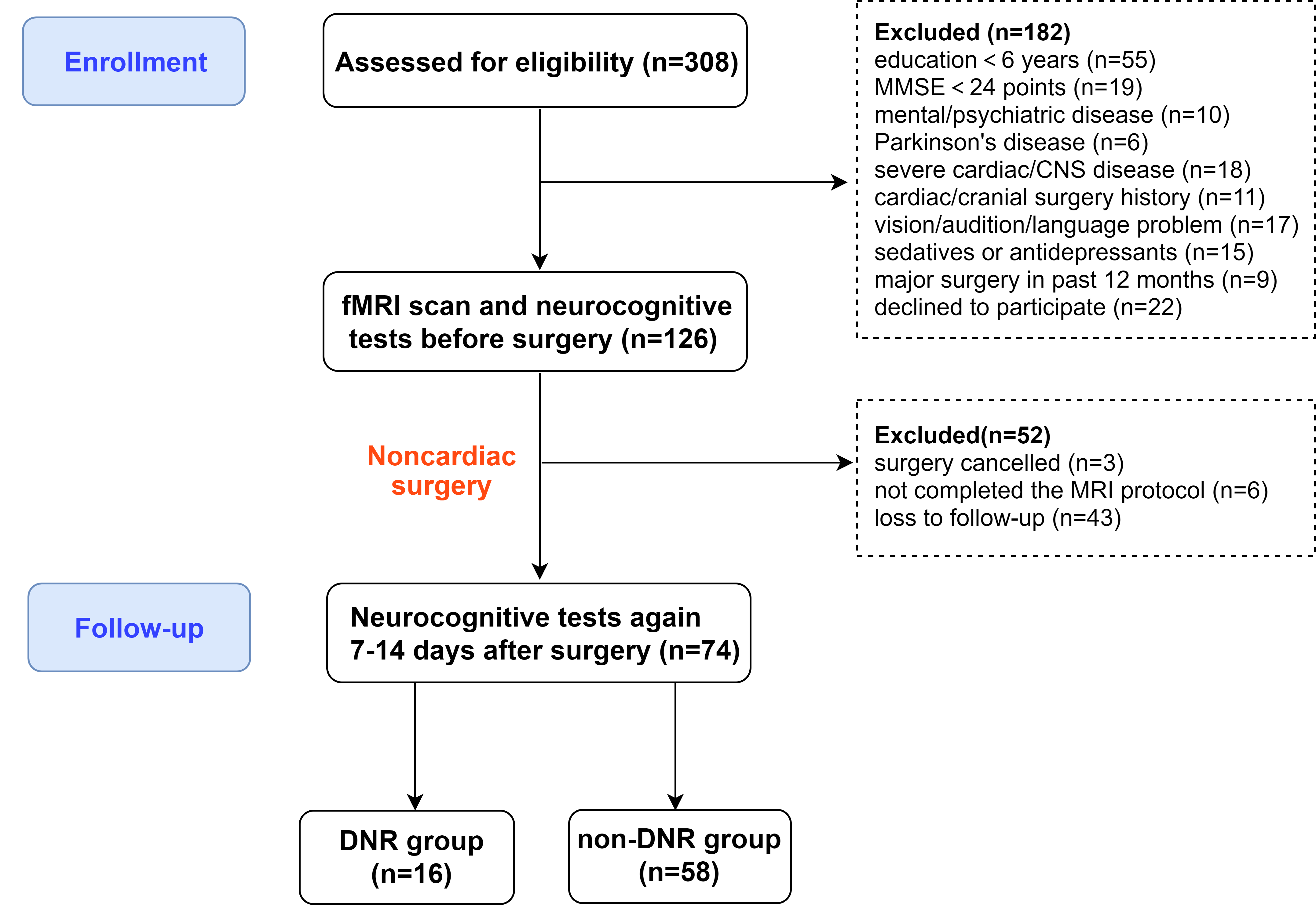


**Supplemental Figure 1. Study design and flowchart.**

Abbreviations: CNS, central nervous system; DNR, delayed neurocognitive recovery; fMRI, functional magnetic resonance imaging; MMSE, mini-mental state examination.


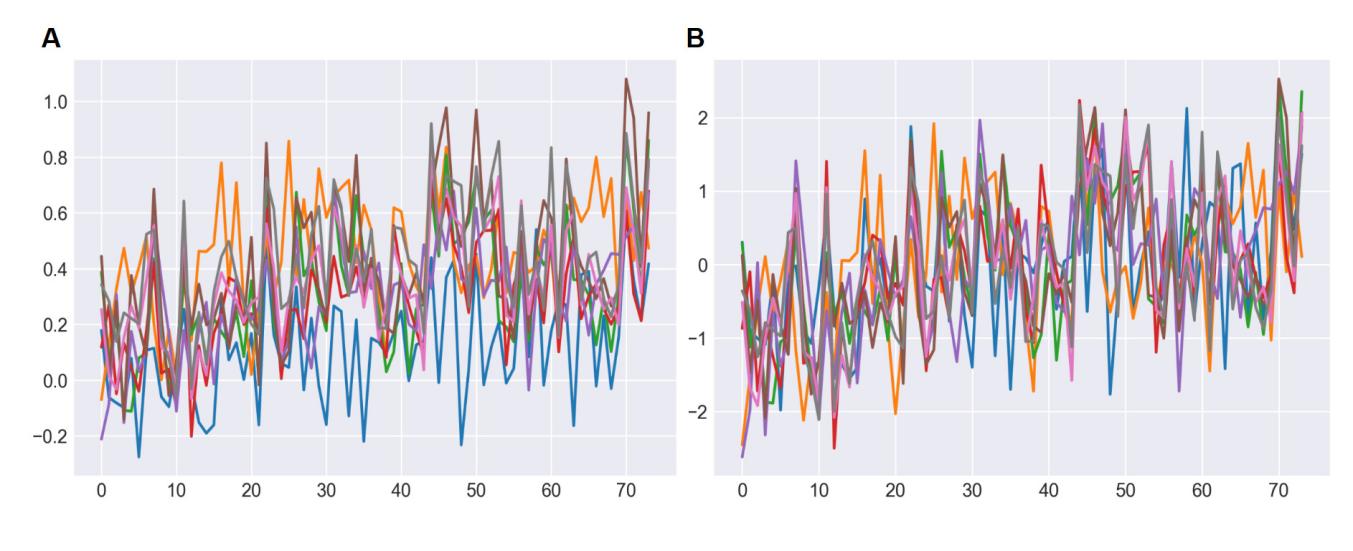


**Supplemental Figure 2. The data distribution of all features. (A)** The raw data; **(B)** the standardized data.
